# Numerical Investigation of Active Flow Control Around a Generic Truck A-Pillar

**DOI:** 10.1007/s10494-016-9760-3

**Published:** 2016-08-30

**Authors:** G. Minelli, S. Krajnović, B. Basara, B. R. Noack

**Affiliations:** 1 Department of Applied Mechanics, Chalmers University of Technology, 412 96 Gothenburg, Sweden; 2 Advanced Simulation Technologies, AVL List GmbH, Hans-List-Platz 1, 8020 Graz, Austria; 3 Institut PPRIME, UPR 3346, Départment Fluides, Thermique, Combustion, CEAT, CNRS Université de Poitiers ENSMA, 43 rue de l’Aérodrome, F-86036 Poitiers, CEDEX France; 4 Institut für Strömungsmechanik, Technische Universität Braunschweig, Hermann-Blenk-Straße 37, D-38108 Braunschweig, Germany

**Keywords:** Active flow control, AFC, Zero net mass flux, Synthetic jet, LES, POD, Truck, A-pillar

## Abstract

Large Eddy Simulations (LES) are conducted to study the actuated flow field around a bluff body. The model is a simplified section of a truck. The aim of the work is to model the separation of the flow acting at the front rounded corners, the so called A-pillars, and to minimize the separation of the flow by means of Zero Net Mass Flux synthetic jets. LES data show the interaction of the flow main structures, the separation mechanism and the effects of the actuation on the flow field. The flow is post processed using modal and frequency decompositions. Relevant results in terms of drag reduction were observed for the actuated flow. The principle flow mechanisms are discussed and an optimal actuation frequency, in terms of induced fluctuations and drag reduction, is identified.

## Introduction

The attempt to reduce the aerodynamic drag in trucks, and more generally in road vehicles, has become a major challenge. The need of reducing fuel consumption and the continuous request for better performance of vehicles has incited research to adapt successful aerodynamic solutions to road transportation. Spoiler and add-ons are nowadays visible on heavy transport vehicle but the shape of a truck is still far from the optimal aerodynamic solution. On the contrary a truck is optimized for cargo and stocking operation. Therefore, the main challenge of today remains to keep the load optimized shape while improving its aerodynamic. The main source of drag in a truck, principally arise from four different regions: the base region, wheels housing and underbody, the gap between tractor and trailer and the front of the tractor. Aerodynamic research has investigated solutions to improve the aerodynamic performances of these regions. For example cab deflectors are widely used to overcome the gap between tractor and trailer. In the early 80’ the sharp front corners of the trailer (the so called A-pillar) were smoothed to rounded. Flaps applied at the trailing edge [1], or other add-ons [2] have been recently investigated in order to delay the separation of the flow and reducing the wake effects. These examples have a key factor in common: they are passive flow control techniques, thus designed to work at a nominal condition which seldom matches with the real operating flow state. Here rises the importance to design a control adaptable to the flow condition, an Active Flow Control (AFC). Indeed, the AFC opens the possibility for feedback control (open-loop) once the closed-loop flow mechanisms are well understood. The AFC can be classified in three main categories: flow control by means of moving surfaces [3], by means of plasma actuators [4], and by means of synthetic jets. The main downsize of the former technique lies in its applicability due to geometrical constrains. The moving surfaces need mechanical transmission and electric engines which are not easy to embed in the original vehicle geometry. Plasma actuator research for flow control started 20 years ago [5] and produced promising results, yet far from being extensively applicable in the near future. For example, more research is needed to understand the degradation of the actuation over time and their power consumption [6]. Thus, the synthetic jets turned out to be the most effective way to manipulate the flow field. The AFC used in this work is a Zero Net Mass Flux (ZNMF) synthetic jet. In comparison with the work of Krajnović et al. [7], the ZNMF does not employ steady sucking or blowing but it moves air by means of an oscillating membrane. Promising results of this technique were found in several studies, for both airfoils [8, 9], bluff bodies [10] and generic vehicles [11]. Their main achievement and potential future development are collected in the reviews provided by Gad-el-Hak [12], by Cattafesta and Sheplak [13] and more recently by Brunton and Noack [14].

**Fig. 1:**
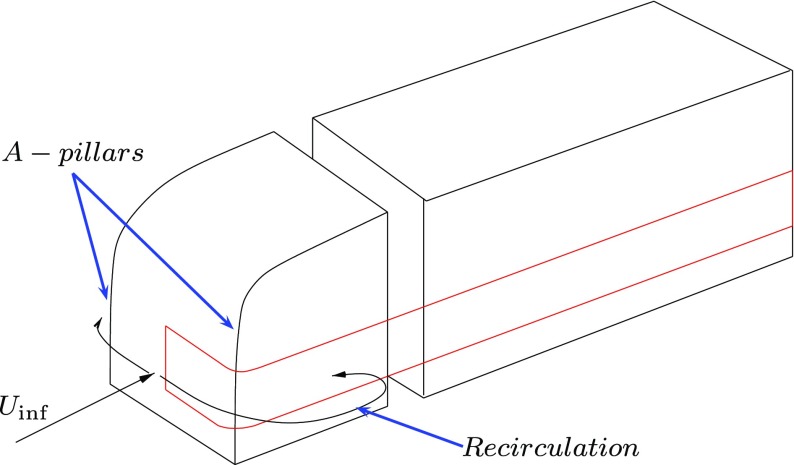
The section of the truck and the natural behaviour of the flow

The ZNMF were successfully used for open-loop [15, 16], and closed-loop studies [17], in order to reduce the drag of bluff bodies acting on the base region. On the contrary, the present work focuses on the separation acting at the front rounded corner of a truck, the so called A-pillar. The type of separation occurring in the present study can be reconnected, to some extend, to the flow behaviour visualized in stalled airfoils by different authors [8, 18–20]. The main features that characterize the topology of this flow are the separated shear layer, the near wake shedding and their interaction. The natural frequency of the shear layer is usually higher compared to the near wake shedding, but the coupling of them can result in a collective interaction during the formation of the vortices as it was observed by Unal and Rockwell in [21]. This interaction changes behaviour when the flow is controlled actively, in particular when the separation of the shear layer is defined by the actuation frequency. The aim of the present study is twofold: to investigate the shear-wake interaction, clarifying how it changes for different actuations, and to use a ZNMF active flow control to delay separation and suppress the separated flow region occurring at the side of a truck, Fig. 1.

Large Eddy Simulations (LES) are employed to resolve large part of the unsteady structures present in the flow and reproduce an unsteady actuation. A modal analysis is also needed to extract the main flow features and structures interactions. LES results were successfully post-processed previously by means of Proper Orthogonal Decomposition (POD) by Östh et al. [22, 23]. Thus, POD is used in this work and a Fast Fourier Transform (FFT) analysis is also made to extract frequencies and compare the results found in the POD.

This paper focuses on the understanding of the separation mechanism occurring at the A-pillar. 3-D effects are not considered, therefore periodic boundary condition which defines an imaginary infinite A-pillar has been used. This allows to study the nature of the flow mechanism, at the same time reducing the computational cost of a well resolved LES. For the same reason LES are performed at a lower Reynolds number compared to the real case. This is a first step toward the study of a realistic A-pillar. Nevertheless, this paper reports a fundamental study regarding the nature of the separation mechanism, achieving the following goals:

- A preliminary study describes the main flow patterns of the unactuated flow.
- The angle formed by the actuation with respect to the local direction of the flow is studied to evaluate the most effective configuration.
- The actuation frequency forces the flow into different flow states. We discuss the effects of such an actuation, identifying an optimal frequency.
- An interpretation of the flow topology of the unactuated and different actuated cases is given.
- A modal decomposition which integrates POD and FFT is used throughout the study to interpret the physics and the interaction of the main flow structures.

## Numerical Set-up

**Fig. 2:**
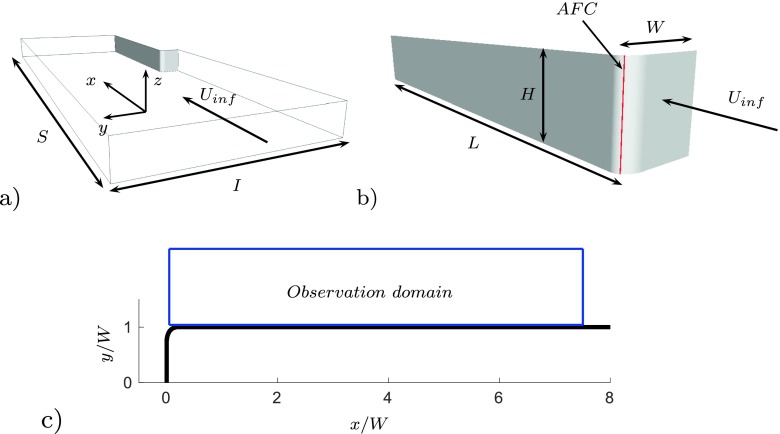
The computational domain, the model and the observed domain

Figure 1 shows a sketch of a simplified truck and the behaviour of the flow at the A-pillar. When the truck moves, with a certain speed *U*_inf_, the flow impinges the front of the tractor and migrates toward the A-pillars. At this point the flow separates creating a recirculation flow region which increases the drag. A section of a truck, highlighted in red in Fig. 1, is significant to study the front separation using a relatively small computational domain. Figure 2 shows the final CFD model and its domain, Fig. 2a.

The reference dimension of the section is, *W* = 1.245*m*, Fig. 2b. The model has a length of *L* = 8*W*, and a height of *H* = 1.12*W*. The length of the domain is *S* = 19.2*W* and the width is *I* = 8*W*. A uniform and steady velocity, *U*_*inf*_ = 0.64*m*/*s*, is applied at the inlet. The resulting Reynolds number based on the double width of the section, 2*W* (width of the entire truck), and *U*_*inf*_ is *R* *e* = 1×10^5^. The curvature of the rounded corner is defined by an spline described by the points listed in Table 1. The actuation slot, which extends along *z* direction, is bounded by the spline points *p*_6_ and *p*_7_, Table 1. A homogeneous Neumann boundary condition is applied at the outlet. The surfaces of the body are treated as no slip walls. Periodic boundary conditions are imposed at the upper and lower walls which define the height, *H*, of the section. Symmetry boundary conditions are set at the two side walls, which define the width, *I*, of the domain. A time varying velocity is set at the actuation slot highlighted in red in Fig. 2b. A finite grid plane, placed at *z* = *H*/2, is used to sample snapshots of the flow field, Fig. 2c.

**Table 1:** *x* and *y* coordinates of points describing the curvature spline

Points	*x* coordinate in m	*y* coordinate in m
*p*_1_	0.000	0.931
*p*_2_	0.009	1.023
*p*_3_	0.023	1.070
*p*_4_	0.041	1.114
*p*_5_	0.065	1.153
*p*_6_	0.099	1.192
*p*_7_	0.108	1.200
*p*_8_	0.141	1.223
*p*_9_	0.190	1.242
*p*_10_	0.222	1.245

The governing LES equations are the spatially implicitly filtered Navier-Stokes equations, where the spatial filter is determined by the characteristic width $\Delta =(\Delta _{1}\Delta _{2}\Delta _{3})^{\frac {1}{3}}$, and Δ_*i*_ is the computational cell size in the three coordinate directions.

1 $$ \frac{\partial \bar{u}_{i}}{\partial t} + \frac{\partial}{\partial x_{j}} \left( \bar{u}_{i} \bar{u}_{j} \right) = -\frac{1}{\rho}\frac{\partial \bar{p}}{\partial x_{i}} + \nu \frac{\partial^{2} \bar{u}_{i}}{\partial x_{j}\partial x_{j}} - \frac{\partial \tau_{ij}}{\partial x_{j}} $$

and

2 $$ \frac{\partial \bar{u}_{i}}{ \partial x_{i}}= 0. $$

Here, $\bar {u}_{i}$ and $\bar {p}_{i}$ are the resolved velocity and pressure, respectively, and the bar over the variable denotes the operation of filtering. The influence of the small scales in Eq. 1 appears in the SGS stress tensor, $\tau _{ij}=\overline {u_{i}u_{j}}-\bar {u}_{i}\bar {u}_{j}$. The algebraic eddy viscosity model, described by Smagorinsky in [24], is employed in this work. The Smagorinsky model represents the anisotropic part of the SGS stress tensor, *τ*_*ij*_ as

3 $$ \tau_{ij}- \frac{1}{3}\delta_{ij}\tau_{kk}=-2\nu_{sgs}\bar{S}_{ij} $$

where the SGS viscosity,

4 $$ \nu_{sgs}=(C_{s}f\Delta)^{2}|\bar{S}| $$

and,

5 $$ \bar{S}=\sqrt{(2\bar{S}_{ij}\bar{S}_{ij})} $$

where

6 $$ \bar{S}_{ij}=\frac{1}{2}\left( \frac{\partial \bar{u}_{i}}{\partial x_{j}}+\frac{\partial \bar{u}_{j}}{\partial x_{i}}\right). $$

The Smagorinsky constant, *C*_*s*_ = 0.1, previously used in bluff body LES [25], is used in the present work. *f*, in Eq. 4, is the Van Driest damping function,

7 $$ f=1 - exp\left( \frac{-n^{+}}{25}\right) $$

where *n* ^+^ is the wall normal distance in viscous units.

**Fig. 3:**
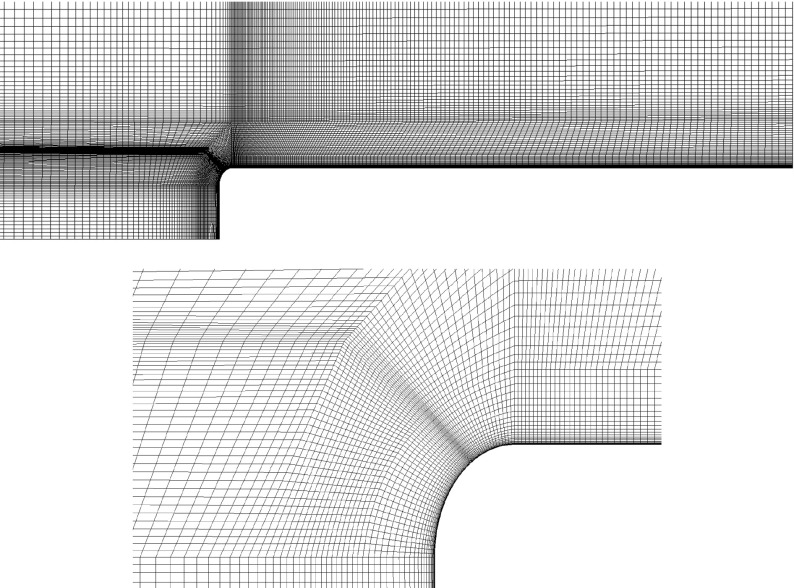
A top view of a plane cut of the computational grid

The simulations in this work are made with the commercial finite volume CFD solver, AVL FIRE [26]. AVL FIRE is based on the cell-centred finite volume approach. The grid topology is constructed using the O-grid technique in order to concentrate most of the computational cells close to the body. Figure 3 shows a cut plane of the grid employed and its refinement at the active flow control region. Pope [27] suggests that a reliable LES grid should resolve the 80 *%* of the turbulent energy. According to Piomelli and Chasnov [28] the first grid point in the wall normal direction must be located at *n* ^+^<1, where $n^{+}=\frac {u_{\tau }n }{\nu }$ with the friction velocity *u*_*τ*_. The resolution in span-wise and stream-wise direction must be Δ*l* ^+^≃15−40 and Δ*s* ^+^≃50−150 respectively, in order to resolve the near-wall structures. Here $\Delta l^{+}=\frac {u_{\tau }\Delta l}{\nu }$ and $\Delta s^{+}=\frac { u_{\tau }\Delta s}{\nu }$. In this work, the grid resolution has an average value in the wall normal direction of *n* ^+^ = 0.45 and a maximum value of *n* ^+^ = 1.7. The resolution in the span-wise direction is Δ*l* ^+^<30, and the resolution in the stream-wise direction is Δ*s* ^+^<150. The chosen time step, Δ*t* ^∗^ = Δ*t* *U*_*inf*_/2*W*, is Δ*t* ^∗^ = 1.28×10^−3^ for all the simulations, resulting in a CFL number lower than 1 in the entire domain. All the simulations were run first until the flow was fully developed. This was followed by an averaging of *t* ^∗^ = *t* *U*_*inf*_/2*W* = 25. The NSE are solved using a collocated grid arrangement. The convective fluxes are approximated by a blend of 96 *%* linear interpolation of second order accuracy (central differencing scheme) and of 4 *%* upwind differences of first order accuracy (upwind scheme). The time marching procedure is done using the implicit second-order accurate three-time level scheme. To determine the pressure, Eq. 2 is converted into an equation for the pressure correction. The well known SIMPLE algorithm is used to update pressure and velocity fields in order to satisfy the continuity equation.

**Table 2:** Details of the computational grids, aerodynamic drag (*C*_*d*_) and side force (*C*_*s*_) coefficients

Case	Fine grid	Middle grid	Coarse grid
Number of cell	4.5 millions	2.1 millions	1.2 millions
$y^{+}_{mean}$	<0.5	<0.5	<0.5
$\Delta l^{+}_{max}$	<20	<30	<50
$\Delta s^{+}_{max}$	<80	<150	<200
*CFL*	<1	<1	<1
*C*_*d*_	0.38	0.38	0.37
*C*_*s*_	7.7	7.7	7.5

### Numerical accuracy

**Fig. 4:**
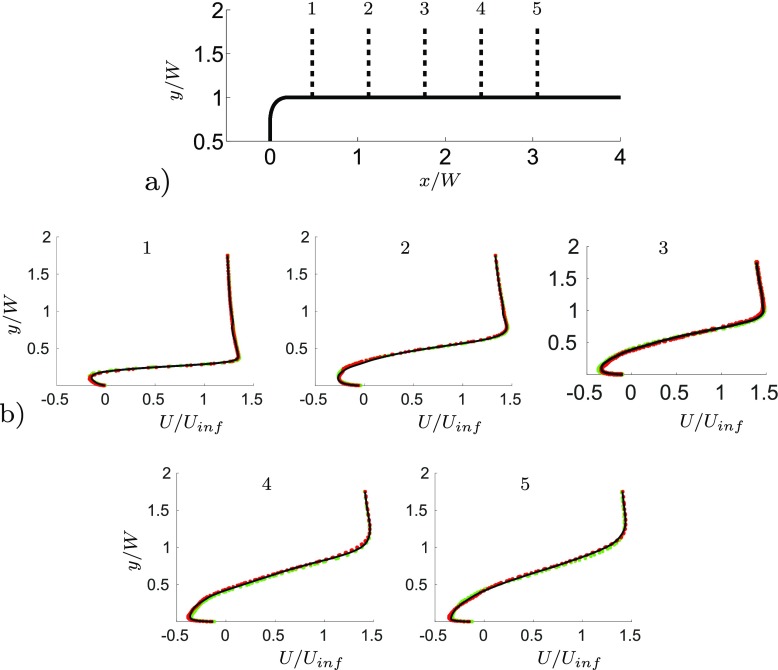
**a** Profiles’ location along the body. *x*_1_/*W* = 0.48, *x*_2_/*W* = 1.12, *x*_3_/*W* = 1.76, *x*_4_/*W* = 2.4, *x*_5_/*W* = 3.04. **b** Averaged streamwise velocity component profiles. Fine grid (*red dotted line*), middle grid (*black solid line*) and coarse grid (*green dotted line*)

**Fig. 5:**
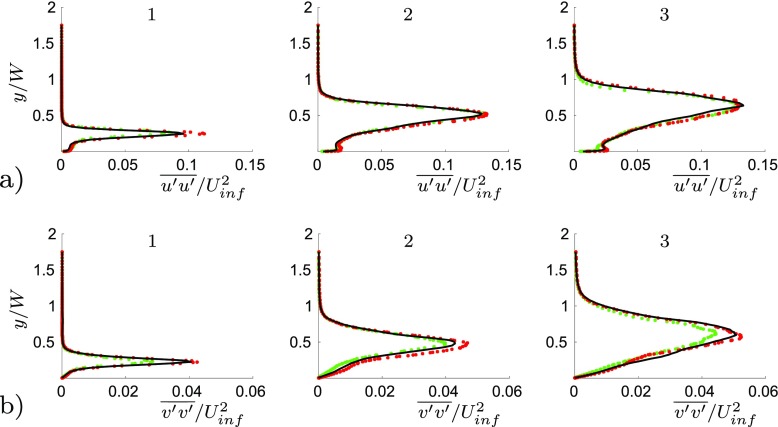
**a** Averaged $\overline {u^{\prime }u^{\prime }}$ stress component profiles. **b** Averaged $\overline {v^{\prime }v^{\prime }}$ stress component profiles. Fine grid (*red dotted line*), middle grid (*black solid line*) and coarse grid (*green dotted line*). The numbers on the single plots refer to Fig. 4a

The numerical accuracy is ensured by performing the unactuated flow field simulation on three computational grids. The details of the meshes are summarised in Table 2. The number of computational cells of the coarse grid has been reduced by 40 *%* with respect the middle grid. The coarsening procedure affects only the streamwise and spanwise directions. Thus, the grid has not been coarsened in the wall normal direction. Table 2 shows the good agreement of the three simulations concerning the drag and side force coefficient. Figure 4a) show the chosen profiles’ locations along the body, while Fig. 4b shows the streamwise velocity profiles of both the fine, the middle and the coarse grids at the chosen locations. The chosen location correspond to the following *x* coordinates: *x*_1_/*W* = 0.48, *x*_2_/*W* = 1.12, *x*_3_/*W* = 1.76, *x*_4_/*W* = 2.4, *x*_5_/*W* = 3.04. Figure 5a and b shows the normalized $\overline {u^{\prime }u^{\prime }}$ and $\overline {v^{\prime }v^{\prime }}$ stresses respectively, at three different locations. The mean value of drag and side force, Table 2, and the good agreement between the streamwise velocity Fig. 4 and stresses Fig. 5 profiles, confirms that mesh independence is achieved. Thus the middle grid has been employed to carry out the study presented in the results section.

### Modal and frequency analyses

One single POD temporal coefficient can oscillate at different frequencies, while the Fast Fourier Transform (FFT) analysis represents the area of interest of the actual frequencies of the flow. It is interesting to compare the two approaches in order to gain a complete understanding of the flow structures in terms of both the energy content and characteristic frequencies.

The present POD is made of a equidistantly sampled pressure snapshots *p* ^*m*^ = *p*(***x***, *t* ^*m*^) at time *t* ^*m*^ = *m*Δ*t*, *m* = 1,..., *M* with the time Δ*t*, and a Cartesian coordinate system ***x*** = (*x*, *y*, *z*) with unit vectors ***e***_*x*_, ***e***_*y*_, ***e***_*z*_ respectively.

As it was originally proposed by Lumley [29], this method is based on energy ranking of orthogonal structures computed from a correlation matrix of the snapshots. A Singular Value Decomposition (SVD) approach is used to conduct the POD analysis on the mentioned set of snapshots. In the present POD, the pressure is decomposed in the mean field, 〈*p*〉, and the fluctuating part, *p* ^′^, as

8 $$ p(\textit{\textbf{x}}, t)=\langle p \rangle (\textit{\textbf{x}})+p^{\prime}(\textit{\textbf{x}},t) $$

The fluctuating part is then approximated, by the SVD approach, with space dependent modes, *p*_*i*_, and time dependent mode coefficient, *b*_*i*_, as

9 $$ p^{\prime}(\textit{\textbf{x}}, t)=\sum\limits_{i=1}^{\infty}b_{i}(t)p_{i}(\textit{\textbf{x}})\approx\sum\limits_{i=1}^{N-1}b_{i}(t)p_{i}(\textit{\textbf{x}})+p_{res}(\textit{\textbf{x}},t) $$

The definition can now be written in a more compact form if we consider that *b*_0_ = 1 and *p*_0_ = 〈*p*〉 following [30],

10 $$ p(\textit{\textbf{x}}, t)=\sum\limits_{i=0}^{N-1}b_{i}(t)p_{i}(\textit{\textbf{x}}) $$

The first and second moments of the POD modes coefficients are:

11 $$ \langle b_{i}\rangle = 0;\;\;\;\;\langle b_{i} b_{j}\rangle = \mu_{i}\delta_{ij} $$

The energy content of the single mode, *K*_*i*_, is approximated from the mode coefficients as

12 $$ K_{i}(t)=\frac{1}{2}{b_{i}^{2}}(t) $$

and the total energy, *K*_Σ_(*t*), is evaluated as

13 $$ K_{\Sigma}(t)=\sum\limits_{i=1}^{N-1}K_{i}(t) $$

**Fig. 6:**
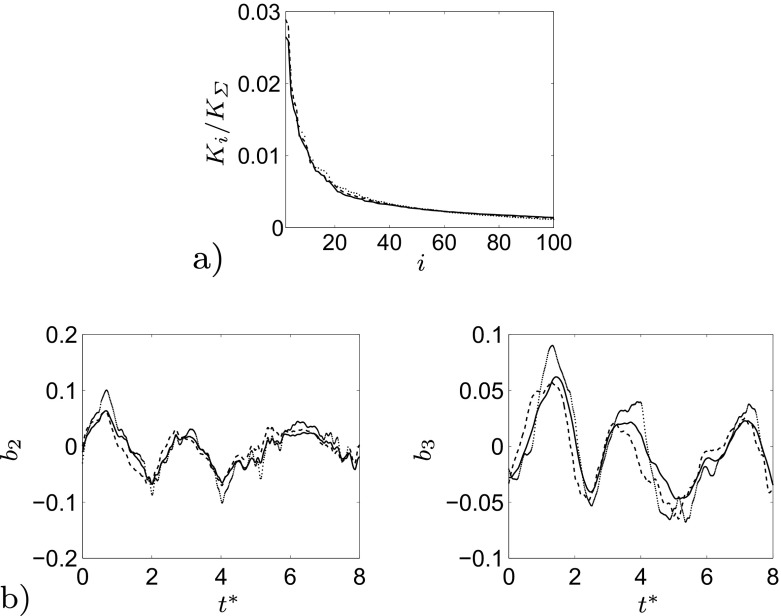
**a** Pressure modes energy content of three different decompositions, from mode 2 to mode 100. **b** Second (*left*) and third (*right*) pressure temporal coefficients for three different decompositions. 1000 snapshots (*black solid line*), 800 snapshots (*black dashed line*), 600 snapshots (*black dotted line*)

In the present work, the POD study is performed over 1000 equispaced time steps and the non dimensional time step Δ*t* ^⋆^ between each snapshot is chosen as Δ*t* ^⋆^ = 1.28×10^−2^. In the present POD, mode 1 represents the mean value of the flow field and it contains the largest amount of energy. Every travelling structure is represented by a couple of modes, containing the same amount of energy but shifted in time. For example the most energetic travelling structures is represented by the sum of mode 2 and 3, visualised as 1^*st*^ *pair* in Fig. 8a. Figure 8b shows the temporal coefficients of mode 2 and 3, which are similar but shifted in time. The shifting is necessary to reconstruct the motion of the considered flow structures. The accuracy of the POD is ensured performing the calculation on three different sets of snapshots, Fig. 6. Both the energy content of the first 100 modes, Fig. 6a, and the time history of the first two temporal coefficients, Fig. 6b, does not differ excessively between the three sets of snapshots. This confirms that the chosen time window covered by the snapshots is large enough to capture the main features of the flow.

### Actuation’s parameters

The magnitude of the velocity at the actuation region (red slot in Fig. 2), *U*_*afc*_, is defined by the time varying boundary condition as follows,

14 $$ U_{afc}=0.1U_{inf}\sin\left( {t2\pi f_{a}}\right) $$

where *U*_*inf*_ is the magnitude of the free stream velocity, and *f*_*a*_ is the chosen actuation frequency. Two non dimensional parameters describe the performances of the actuation. The first parameter is the momentum coefficient *C*_*μ*_. It is an indicator of the energy spent for the actuation with respect to the energy of the unactuated flow.

15 $$ C_{\mu}=\frac{W_{afc}U_{afc}^{2}}{WU_{inf}^{2}}=1\times10^{-4}. $$

Here *W*_*afc*_ = 0.012*m* is the width of the actuation slot. *C*_*μ*_ has a low value but sufficient to excite the thin boundary layer that characterizes the attached flow before the separation. All the frequencies in the present work are described in terms of the second non dimensional parameter, the reduced frequency *F* ^+^ (also called actuation Strouhal number).

16 $$ F^{+}=\frac{f}{U_{inf}/L_{rec}} $$

**Fig. 7:**
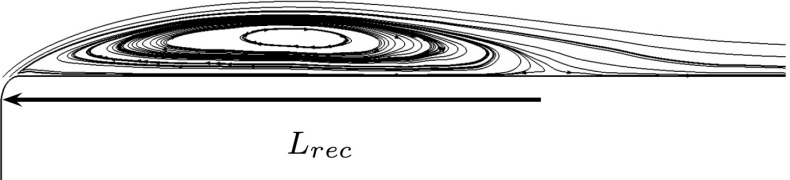
Streamlines of the streamwise averaged velocity field projected on a middle cut plane. *L*_*rec*_ is the length of the recirculation bubble for the unactuated flow field. Flow from left to right

Here *f* represents the frequency in Hertz and *L*_*rec*_ = 6.5*m* is the measured length of the recirculation bubble measured in the unactuated flow, Fig. 7.

## Results

A preliminary study to evaluate the main features of the unactuated flow field is presented in the first part of this section. Results from both unactuated and actuated flows at different actuation frequencies are compared in the second part.

### Preliminary study. the unactuated flow

The unactuated flow field was first studied to get a better understanding of its main features.

**Fig. 8:**
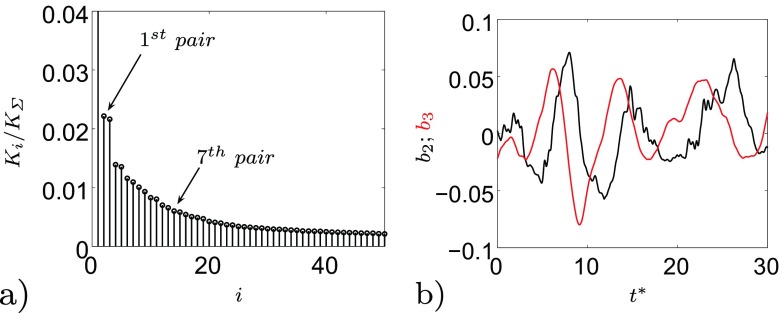
**a** Energy content of the first 50 most energetic pressure modes. **b** Comparison of the temporal coefficient *b*_2_ (*black*) and *b*_3_ (*red*). Unactuated flow

**Fig. 9:**
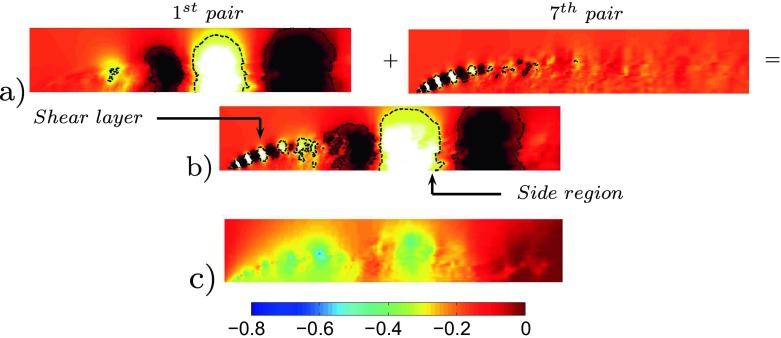
**a** Spatial distribution of the 1^*st*^ (*left*) and 7^*th*^ (*right*) pair of modes’ travelling structures. **b** A reconstruction of the pressure field using the 1^*st*^ and 7^*th*^ pair of modes. **c** A snapshot of the relative pressure flow field, in [Pa]. Flow from left to right

**Fig. 10:**
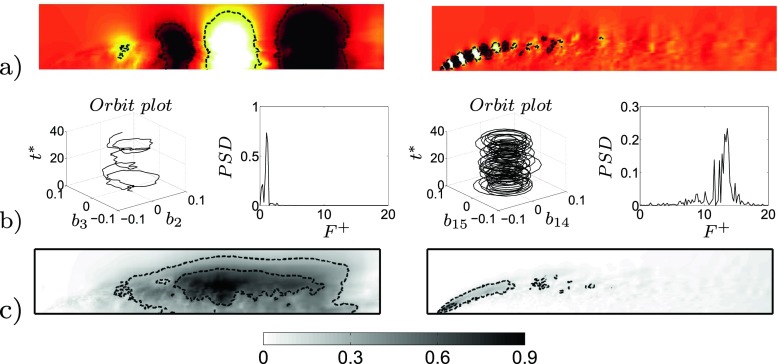
**a** Spatial distribution of the 1^*st*^ (*left*) and 7^*th*^ (*right*) pair of modes’ travelling structures. **b** Pair 1, temporal coefficient’s orbit plot and PSD (*left*), and pair 7, temporal coefficient’s orbit plot and PSD (*right*). **c** Spatial distribution of *F* ^+^ = 1.4 and *F* ^+^ = 13, from FFT analysis. Flow from left to right

The POD performed on the unactuated flow is able to reproduce the main features of the flow. Figure 8a shows the energy content of the first 50 pressure modes with respect to the considered time interval. Mode 1 corresponds to the averaged flow field and it is the mode that contains the most energy. Figure 8a shows also that neighbouring modes contain the same amount of energy in pairs. Each pair of modes, with the exclusion of mode 1, describes the characteristic travelling structures acting in the flow domain. Figure 9a and b depicts a reconstruction of the pressure field by characteristic modes in comparison with the instantaneous pressure flow field, Fig. 9c. Figure 9 clarifies the conduct of the flow mentioned in the introductory section and widely described in [21]. Both the small structures at the front and the larger structures at the side are captured by two different pairs of modes, Fig. 9a. In merging the modes, a reconstruction in both time and space of the flow structures is observed, Fig. 9b. The POD separates the eddies which describe the detached shear layer (pair 7) from the eddies of the side region (pair 1). The shear layer eddies contain a much smaller energy content but they are fundamental for the dynamic of the flow. As it will be described in the following section, the actuation acts directly on the separated shear which can drastically shape the separated flow region. An FFT analysis conducted on the same set of snapshots indicates the spatial distribution of the most important frequencies describing the flow field. FFT is applied to each cell of the observed domain (Fig. 2c). Figure 10 highlights the differences in energy and space distribution of two different flow structures. The POD results (Fig. 10a and b) are compared against the FFT results, Fig. 10c. Figure 10c clearly shows that structures moving at *F* ^+^ = 1.4 have a larger energy content and space distribution (side region structures), while *F* ^+^ = 13 is only visible in a limited area and its energy is considerably lower (separated shear layer eddies). Figure 10b shows the PSD and the orbit plot of the corresponding temporal modes. In particular the orbit plot describes the time history of the time coefficients and highlights their possible periodicity. The periodicity of the time coefficients is strong and remarks the regular recurrence of the described structures. The spatial distribution of POD structures (Fig. 10a) and the frequency of its temporal mode (Fig. 10b) match well with the spatial distribution of the corresponding frequency extracted from the FFT analysis (Fig. 10c).

**Fig. 11:**
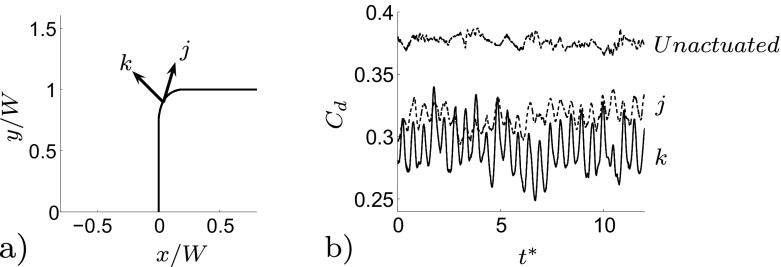
**a** Two different actuation configurations. *k*, the direction of the actuation velocity *U*_*afc*_ is normal to the local surface and consequentially perpendicular to the local direction of the flow. *j*, the direction of the actuation velocity *U*_*afc*_ forms an angle of 20^∘^ with respect to the tangent of the model’s surface, flow from left to right. **b** *C*_*d*_ time history for the unactuated and actuated flow field

**Fig. 12:**
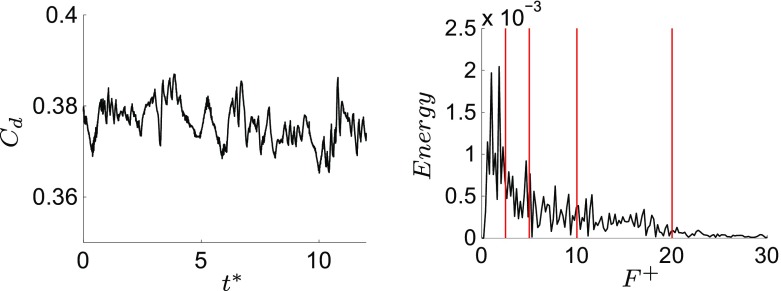
*C*_*d*_ of the unactuated flow (*left*) over time. FFT of *C*_*d*_ (*right*). The red lines represent the chosen actuation frequencies

Before studying the effect of the actuation frequency on the flow, different directions of the actuation are tested. The normal actuation with respect to the local direction of the flow, direction *k* shown in Fig. 11a, is the most effective in terms of drag reduction, Fig. 11b. When the direction of the actuation forms an angle of 20^∘^ with respect to the tangent of the model’s surface, direction *j* the performance are worsen, in particular the mean value of the drag coefficient increases with respect to the previous direction *k*. Thus, the normal actuation was kept for all the actuated configurations. The results shown so far describe the main features of the unactuated flow. Two main dynamics depict the flow: the separated shear layer eddies which move at a high frequency and the side region which contains low frequency structures (highlighted by the 7^*th*^ *p* *a* *i* *r* and 1^*st*^ *p* *a* *i* *r* of modes respectively, Fig. 9a). Even if the energy content of the first group of eddies is very low compared to the energy of the second one, their dynamic affects strongly the side region as will be explained in the following section. The drag coefficient history, Fig. 12a and its FFT plot, Fig. 12b, highlight significant frequencies in a range from *F* ^+^ = 0.6 to *F* ^+^ = 30. Thus, four different configurations are selected for the actuation: case 1 actuated at *F* ^+^ = 2.5, case 2 at *F* ^+^ = 5, case 3 at *F* ^+^ = 10 and case 4 actuated at *F* ^+^ = 20.

### The actuated flow

**Fig. 13:**
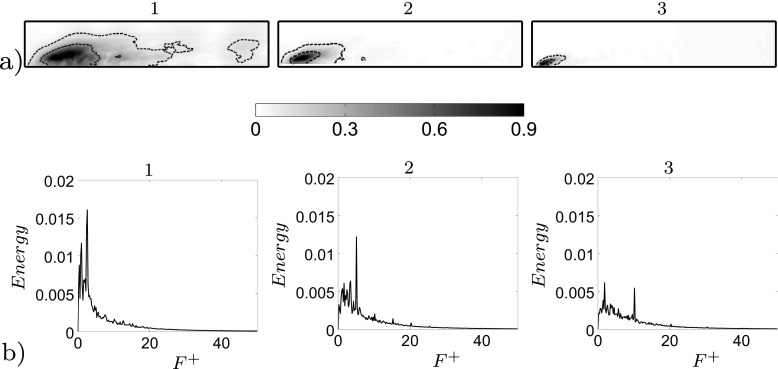
**a** Spatial distribution of the energy of the actuation frequency for each case, flow from left to right. **b** Averaged value of the FFT over the observed domain. From left to right, case 1, case 2 and case 3

**Fig. 14:**
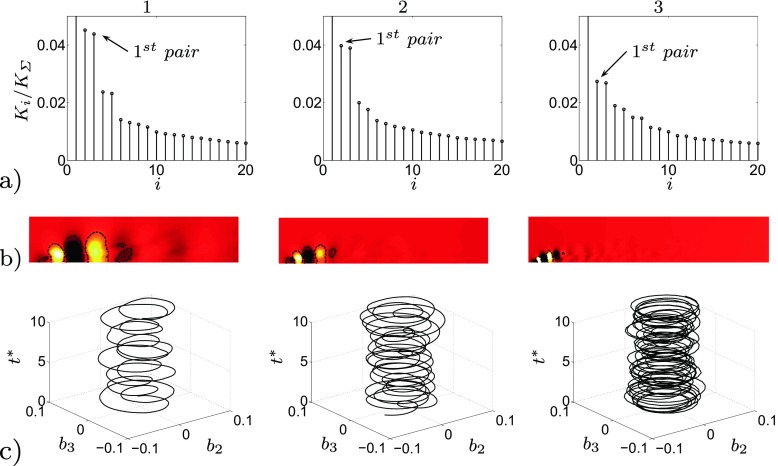
**a** Energy content of the first 20 pressure modes. **b** Spatial distribution of the most energetic mode, flow from left to right. **c** Orbit plot of the corresponding temporal coefficients. From left to right, case 1, case 2 and case 3

**Fig. 15:**
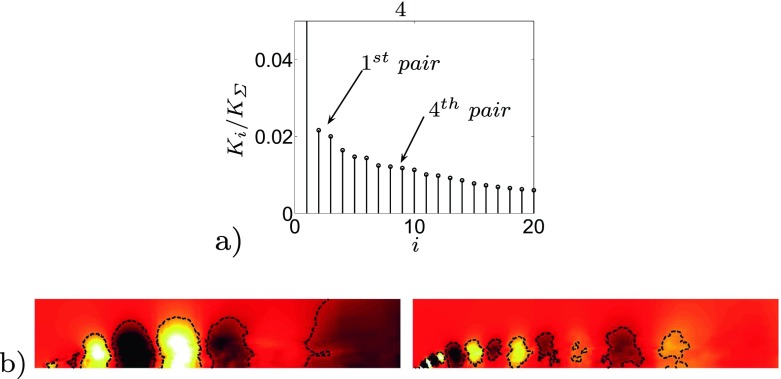
Case 4 POD structures. **a** Energy content of the first 20 pressure modes. **b** Spatial distribution of the most energetic mode (*left*) and the 4^*th*^ pair (*right*), flow from left to right

**Fig. 16:**
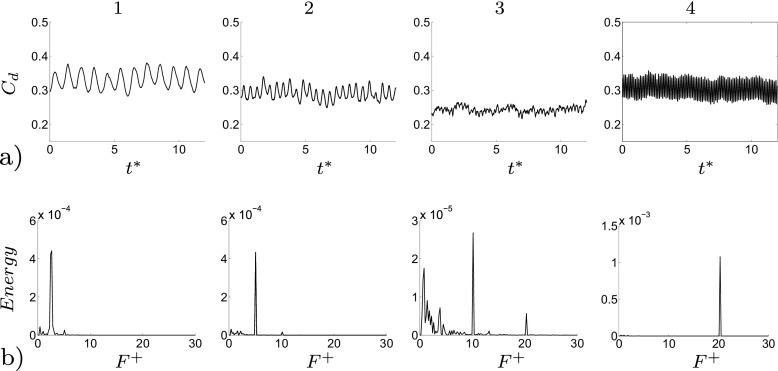
**a** *C*_*d*_ time history. **b** FFT of *C*_*d*_. From left to right, case 1, 2, 3 and 4

**Fig. 17:**
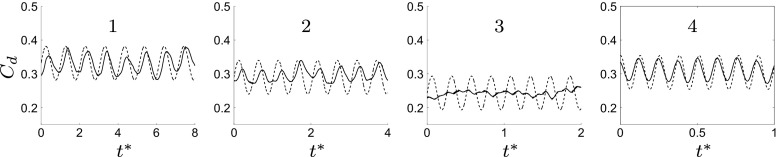
*C*_*d*_ (*black solid line*) and the actuation signal (*black dashed line*) over time. From left to right, case 1, 2, 3 and 4

**Fig. 18:**
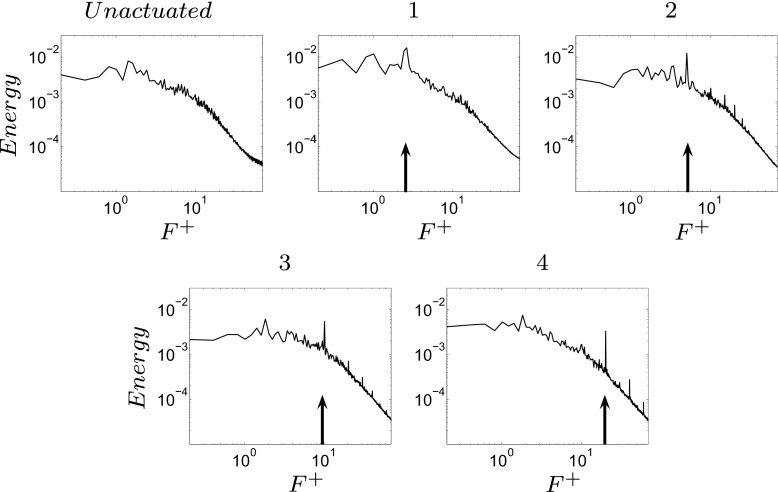
Averaged global energy level of the pressure flow field over frequencies. The black arrows indicate the actuation frequency for each case

**Fig. 19:**
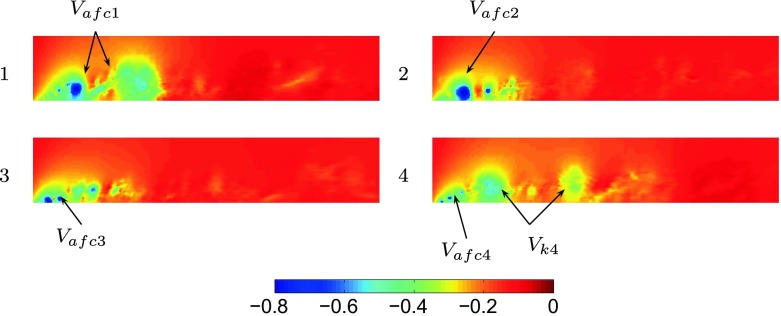
Instantaneous relative pressure field for each actuated case, in [Pa]. Flow from left to right

**Fig. 20:**
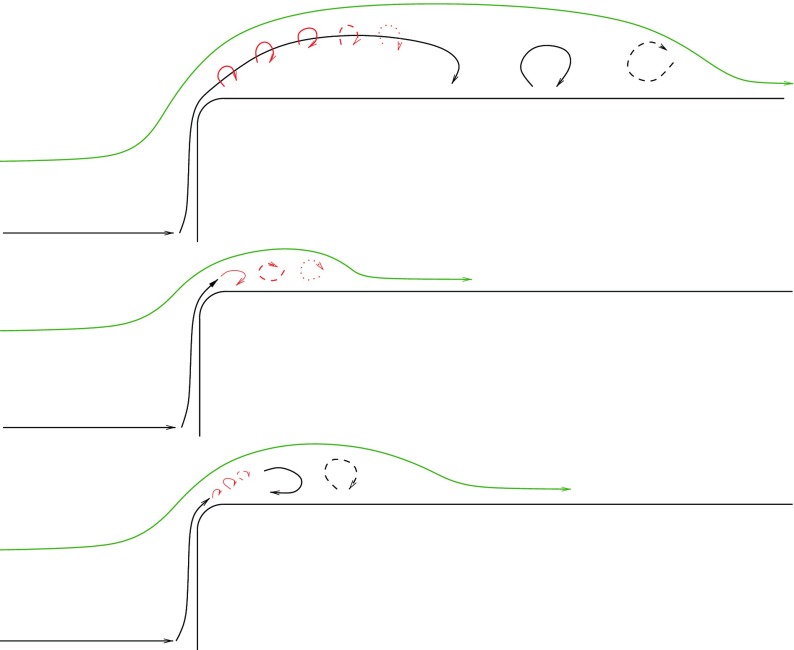
Flow topology. From top, unactuated flow, case 3 and case 4. Shear layer eddies in red and side region structures in black. Streamline of the averaged flow velocity in green. Flow from left to right

**Fig. 21:**
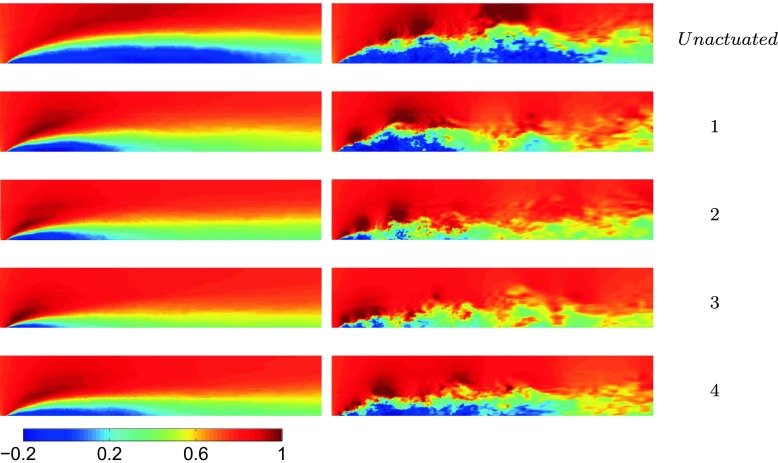
Recirculation bubble reduction. Averaged streamwise velocity component (*left*) and instantaneous streamwise velocity component (*right*), in [m/s]. Flow from left to right

**Fig. 22:**
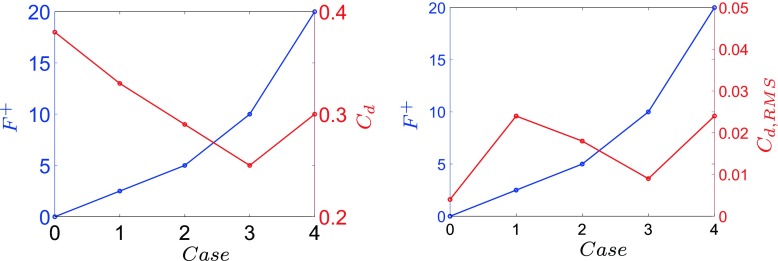
*C*_*d*_ and its RMS trends (*red solid line*) with increasing actuation frequency (*blue solid line*)

An optimal actuation is found in terms of induced fluctuations and drag reduction. The actuation introduces artificial oscillations in the flow, visible from the Root Mean Square (RMS) values of the drag, Table 3. The maximum drag reduction and minimum induced oscillations are observed for case 3, Table 3. Nevertheless, a deeper study of the instantaneous flow field is needed to gain a better understanding of the mechanisms that lead to the optimal configuration. Figure 13 shows how the actuation frequency affects the flow around the body. Figure 13a shows the spatial distribution of the actuation frequency for each case, respectively. The area characterized by the actuation frequency decreases, increasing the actuation frequency. Figure 13b shows the averaged value of the flow energy over the observed domain. Cases 1 and 2 show a clear peak defined by the actuation frequency. The energy of the actuation decreases for case 3 with respect to lower frequencies of the flow; the broader spectrum of case 3 defines that the actuation frequency is not clearly dominant. Noteworthy is also the drastic decrease of the overall flow energy in case 3 (see also Fig. 18 and Table 4). The POD results in Fig. 14 show the same results as shown in Fig. 13. Increasing the actuation frequency, the first most energetic mode decreases its area of interest, Fig. 14b, and its energy content in comparison to the other modes, Fig. 14a. Figure 14c shows the orbit plot of the corresponding temporal coefficients for cases 1, 2 and 3. All cases but the fourth, present a strong periodicity of the first pair of modes defined by the actuation frequency. In case 4, Fig. 15, the first pair of modes does not represent the actuation frequency as for the previous cases. The most energetic structures are large and defined by a low frequency (*F* ^+^ = 2). Only in the 4^*th*^ pair is visible a trace of the separated shear layer structures defined by *F* ^+^ = 20 (the actuation frequency). The signal of the coefficient of drag, *C*_*d*_, depicts the trend of the drag, increasing the actuation frequency (Fig. 16). Figure 16 shows *C*_*d*_ and its FFT for four different actuated cases. As was mentioned before, an optimal actuation frequency is found and shown in Fig. 16. The increase in the actuation frequency does not always benefit the aerodynamic performance of the model. In particular, case 4 shows an increase in both the induced fluctuation and mean value of the drag. Figure 16b shows how the induced fluctuation has less energy compared to the lower fluctuations of the flow. As was observed in Fig. 13b, a broader spectrum of characteristic frequencies is visible in Fig. 16b case 3, while all the other cases present a distinct peak at the actuation frequency. Figure 17 shows a zoom of the coefficient of drag in comparison with the sinusoidal actuation signal. The actuation signal represents the ZNMF actuation. In Fig. 17 the mean value of the actuation signal (dashed line) is shifted from the real zero mean value in order to compare it to the drag coefficient. Figure 17 clearly shows the difference in the dependency of the drag from the actuation. *C*_*d*_ of cases 1 and 4 shows a strong dependency from the actuation. *C*_*d*_ of case 2, and especially case 3, shows a weaker dependence from the actuation. In the latter case, the actuation is not able to force the drag signal following its oscillations, benefitting the overall performance. Furthermore, the integral energy level of the pressure flow field increases from case 3 to case 4, Fig 18 and Table 4. Figure 18 and Table 4 show how the 4^*th*^ actuation brings more energy to lower frequencies of the flow, increasing the integral level of energy, thus the mean drag value. This seems to be in contrast with the results in Figs. 14 and 15. Previously was shown that an increase of *F* ^+^ is followed by a decrease of the influence of the actuation on the overall flow. In particular the energy of the mode representing the actuation decreases by increasing *F* ^+^. On the other hand the energy is not the only parameter that defines a flow. The dynamic of the interaction between the shear layer and the side region structures clarifies the elusive result of case 4 found in the *C*_*d*_ analysis. Figure 19 shows the instantaneous pressure field for the four different configurations. The low pressure areas at the front part indicate the cores of the Kelvin-Helmotz vortices that arise after the separation at the A-pillar. All *V*_*afci*_ are forced by the actuation which defines their frequency, position and dimension. Cases 1 and 2 present larger vortex cores. In these configurations, the vortex has time to develop into larger structures (between an actuation period and its subsequent), *V*_*afc*1_ and *V*_*afc*2_. The first three cases present a definite trend; increasing the frequency, the dimension of *V*_*afci*_ decreases, and the vortex remains more attached to the surface of the body. As it was discussed in the preliminary study section, the flow topology is depicted by the motion of the separated shear layer which interacts with the side region larger eddies. The actuation intervenes directly on the shear layer and defines the frequencies and dimensions of its eddies. In the first three cases the interaction is strong, the actuation is dominant and the shear layer forces the side region to lock in with its own frequency. In other words the eddies forming the shear layer and the side region move at the same frequency (forced by the AFC), while affecting a smaller portion of fluid region moving from case 1 to case 3. Case 4 eludes from the described path. Two different eddies are visible: *V*_*afc*4_, which defines the shear with the actuation frequency and a larger eddy *V*_*k*4_, responsible for the increase of the drag’s mean value visualized in Fig. 16. In the latter case the shear layer eddies are (as for the unactuated flow) separated by dimension and frequency from the side region structures. Indeed, the shear does not lock in the side region eddies. In this case, large oscillations, comparable with case 1, are visible in the *C*_*d*_ signal. The oscillations in case 1 are introduced by the large actuation period that allows *V*_*afc*1_ to separate and develop periodically. The core of *V*_*afc*1_ remains close to the surface of the model, yet being large. In case 4 instead, the high momentum that is introduced forces the flow to separate and reattach at the actuation slot, characterizing the drag oscillations. *V*_*afc*4_ is smaller than *V*_*afc*1_ but at the same time more distant from the surface. This fact allows the formation of a new and larger vortex, *V*_*k*4_, that characterizes the mean value of the drag, widening the recirculation bubble, Figs. 20 and 21. An interpretation of the flow topology is given in Fig. 20. In the unactuated case the shear layer eddies (red) are visible after the separation, and the side region contains large structures (black) responsible of the large separation bubble (green). In case 3 the shear layer is able to lock in the motion of the side region avoiding the formation of larger structures. The last case, case 4, presents smaller eddies at the shear layer which are not able to sustain the lock in and allow the rise of larger structures which widen the recirculation bubble. Both instantaneous and averaged streamwise velocity flow fields are presented in Fig. 21. The reduction of the recirculation bubble is clearly in favour of case 3. Case 4 shows that further increase of actuation frequency, beyond that in case 3, is not beneficial. Indeed, Fig. 22 describes the trend of the flow behaviour increasing the actuation frequency. The actuation frequency is increased from case 1 to 4, (blue line in Fig. 22), and both *C*_*d*_ and *C*_*d*, *RMS*_ trends show a minimum for case 3. In particular, a drag reduction of 34 % with respect to the unactuated case is observed in case 3. The *C*_*d*, *RMS*_ value evaluated in case 3 decreases by 62 % with respect to the drag fluctuations of case 1.

**Table 3:** Mean *C*_*d*_ and its Root Mean Square values

Case	*C*_*d*_	*C*_*d*, *RMS*_
Unactuated flow	0.38	0.004
1. Actuated flow at *F* ^+^ = 2.5	0.33	0.024
2. Actuated flow at *F* ^+^ = 5	0.29	0.018
3. Actuated flow at *F* ^+^ = 10	0.25	0.009
4. Actuated flow at *F* ^+^ = 20	0.30	0.024

**Table 4:** Integral energy value of the pressure flow field

Case	Integral pressure energy
Unactuated	0.18
1. *F* ^+^ = 2.5	0.26
2. *F* ^+^ = 5	0.21
3. *F* ^+^ = 10	0.16
4. *F* ^+^ = 20	0.18

Noteworthy is the connection found between the actuation frequencies and the frequency of the unactuated shear layer. When the actuation has a frequency lower than the unactuated shear layer, the latter is able to lock in the structures of the side region with its own frequency, obtaining a better result the closer is the actuation to the unactuated shear frequency (*F* ^+^ = 13). On the other hand, actuating with a higher frequency (*F* ^+^ = 20) the shear layer cannot sustain the lock in and the overall performances worsen.

## Conclusion

LES at *R* *e* = 1×10^5^ were conducted to analyse the actuation of the flow field around a generic truck A-pillar. Different configurations of the actuation were tested. A preliminary study shows that the actuation is most efficient when the blown and sucked air from the slot is perpendicular to the local direction of the flow. The modal decomposition and the frequency analysis were used to describes the flow field and to extract its main characteristics. The unactuated flow is mainly defined by two eddy patterns. The separated shear layer and the side region structures. The latter contains the most energy present in the flow, while the former is found to play a crucial role in the dynamic of the flow, yet being much less energetic. POD and FFT are used successfully to extract and understand the main flow features. Four actuation frequencies were chosen to study the effects on drag reduction, induced fluctuations and on the dynamic of the flow. The instantaneous and averaged pressure and velocity fields are studied to get a better understanding of the separation mechanism subjected to different actuations. It was furthermore found that, the aerodynamic performances are strongly affected by the actuation, and the study shows the presence of an optimal configuration found in case 3. Case 4 diverts from the performance trend of the previous cases, giving interesting results on the separation features. The closer the actuation frequency is tuned to the frequency of the shear layer of the unactuated case the better performances were achieved, experiencing a worsening when this value was overtaken (case 4). The overall aerodynamic performance of the model benefits when the drag signal becomes independent from the actuation. Both RMS and the mean value of the drag coefficient show a minimum at the optimal configuration. In particular, a drag reduction of 34 *%* is observed in case 3. Finally an interpretation of the flow topology and how the shear layer affect the side region was discussed.
